# Characterizing Key Drivers of Respite Use Among Veteran Caregivers

**DOI:** 10.1093/geroni/igaf122.2975

**Published:** 2025-12-31

**Authors:** Melissa Harris-Gersten, Colleen Blue, Susan Hastings, Zhen Li, Jeanie Lo, Nadya Majette, Jennifer Zervakis, Megan Shepherd-Banigan

**Affiliations:** Durham VA Healthcare System, Durham, North Carolina, United States; Durham VA Health Care System, Durham, North Carolina, United States; Durham VA Healthcare System, Durham, North Carolina, United States; Durham VA Healthcare System, Durham, North Carolina, United States; Veterans Health Administration, Menlo Park, California, United States; Durham VA Healthcare System, Durham, North Carolina, United States; Durham VA Healthcare System, Durham, North Carolina, United States; Duke University, Durham, North Carolina, United States

## Abstract

Respite care has been shown to reduce caregiver burden and may delay long-term care placement, yet it remains underutilized, with only 12-15% of US caregivers reporting use of these services. Understanding drivers of respite use is critical to informing initiatives to improve access. This convergent mixed-methods study aimed to characterize factors influencing respite use among caregivers and Veterans enrolled in the Veterans Health Administration’s (VHA) Caregiver Support Program. A retrospective analysis used machine learning (random forests) to analyze caregiver survey data (2018–2021) linked to Veteran electronic health records (N = 1,727), ranking Veteran and caregiver factors associated with respite use. Semi-structured interviews were conducted with a separate sample of caregivers (N = 20) in 2024, with transcripts analyzed using directed content analysis. Data were integrated using Andersen’s Behavioral Model for Health Service Use. Top predictors included Veteran’s functional limitations, age, health risk, and caregiver’s age and caregiver perceived integration of care with the clinical team. Enabling factors included in-home care support and financial resources; need factors encompassed caregiver burden, depression, loneliness, and Veteran’s chronic conditions, dementia, and frailty. Qualitative findings reinforced the importance of Veteran and caregiver needs while highlighting how VHA’s respite payment model alleviates financial strain. Caregivers described psychosocial and system-level factors, including lack of awareness, concerns about access and quality, and scheduling barriers as reasons not to use respite. Findings provide actionable insights for improving respite uptake by enhancing caregiver-clinician communication, tailoring services, and increasing outreach. Future research should examine how these factors impact respite use in non-VHA settings.

